# Identity and diversity of blood meal hosts of biting midges (Diptera: Ceratopogonidae: *Culicoides* Latreille) in Denmark

**DOI:** 10.1186/1756-3305-5-143

**Published:** 2012-07-23

**Authors:** Sandra B Lassen, Søren Achim Nielsen, Michael Kristensen

**Affiliations:** 1 Department of Agroecology, Aarhus University, Forsøgsvej 1, DK-4200, Slagelse, Denmark; 2 Department of Environmental, Social and Spatial Change, Roskilde University, Universitetsvej 1, DK-4000, Roskilde, Denmark

**Keywords:** COI barcoding, Bluetongue virus, Schmallenberg virus, Blood meal host, *Culicoides*, Ornithophilic insects, Mammalophilic insects

## Abstract

**Background:**

Host preference studies in haematophagous insects e.g. *Culicoides* biting midges are pivotal to assess transmission routes of vector-borne diseases and critical for the development of veterinary contingency plans to identify which species should be included due to their risk potential. Species of *Culicoides* have been found in almost all parts of the world and known to live in a variety of habitats. Several parasites and viruses are transmitted by *Culicoides* biting midges including Bluetongue virus and Schmallenberg virus. The aim of the present study was to determine the identity and diversity of blood meals taken from vertebrate hosts in wild-caught *Culicoides* biting midges near livestock farms.

**Methods:**

Biting midges were collected at weekly intervals for 20 weeks from May to October 2009 using light traps at four collection sites on the island Sealand, Denmark. Blood-fed female biting midges were sorted and head and wings were removed for morphological species identification. The thoraxes and abdomens including the blood meals of the individual females were subsequently subjected to DNA isolation. The molecular marker cytochrome oxidase I (COI barcode) was applied to identify the species of the collected biting midges (GenBank accessions JQ683259-JQ683374). The blood meals were first screened with a species-specific cytochrome b primer pair for cow and if negative with a universal cytochrome b primer pair followed by sequencing to identify mammal or avian blood meal hosts.

**Results:**

Twenty-four species of biting midges were identified from the four study sites. A total of 111,356 *Culicoides* biting midges were collected, of which 2,164 were blood-fed. Specimens of twenty species were identified with blood in their abdomens. Blood meal sources were successfully identified by DNA sequencing from 242 (76%) out of 320 *Culicoides* specimens. Eight species of mammals and seven species of birds were identified as blood meal hosts. The most common host species was the cow, which constituted 77% of the identified blood meals. The second most numerous host species was the common wood pigeon, which constituted 6% of the identified blood meals.

**Conclusions:**

Our results suggest that some *Culicoides* species are opportunistic and readily feed on a variety of mammals and birds, while others seems to be strictly mammalophilic or ornithophilic. Based on their number, dispersal potential and blood feeding behaviour, we conclude that *Culicoides* biting midges are potential vectors for many pathogens not yet introduced to Denmark.

## Background

The importance of biting midges (Diptera, Ceratopogonidae, *Culicoides* Latreille) as medical and veterinary vectors is well established. Several animal and human parasites such as bird and lizard haematoza, horse onchocercosis, wild animal filariasis in both mammals and birds [1,2] and mansonelliasis in humans [3] are transmitted by biting midges. A wide range of arboviral diseases are also transmitted by biting midges [4], such as oropouche virus (OROV), which infects humans, African horse sickness (AHSV), epizootic haemorrhagic disease (EHD) and the closely related bluetongue virus (BTV), which affects wild and domestic ruminants. The latter has spread through large parts of Europe in recent years (2006–09). Currently the Schmallenberg virus (which infects ruminants) is spreading in Northern Europe [5] and has been identified in biting midges from Denmark [6]. These diseases are often associated with the subtropics and have been accidentally introduced into Europe by migration of vectors or transport of infected livestock [4,7].

Species of *Culicoides* have been found in almost all parts of the world except the extreme Polar Regions and some island groups. About 1,400 species are known to live in a wide variety of habitats ranging from sea level to mountainous environments up to 4,000 m and from tropical to high arctic and subarctic regions [4]. Forty-three *Culicoides* species have so far been recorded in Denmark [8].

The host range of biting midges remains largely un-described, but recent studies [9-13] indicate that biting midges acquire blood from a diverse range of mammals and birds, depending upon the relative number and availability of vertebrate hosts. Identification of the origin of blood meals is essential for the determination of host range and host preference of insect vectors. Thus, this study includes a field collection made at farms chosen for their diversity of potential blood meal host species due to both their livestock and location near a forest. Most *Culicoides* species are mammalophilic or ornithophilic, although some feed on reptiles and frogs [14]. The majority of biting midges are exophagous feeders and do not attack their hosts in enclosed places [14]. However, endophagy has been demonstrated in some mammalophilic midge species, which feed on animals inside barns [10,15]. The longevity of adult biting midges has not been determined precisely; it is suggested to last from a few weeks up to several months where recurrent blood meals occur [16]. The recurring need for blood meals creates the unfortunate situation whereby biting midges can transmit various pathogens, e.g. the above-mentioned bluetongue virus among ruminants. Thus, knowledge of the blood feeding behaviour of *Culicoides* biting midges is an essential element in assessing their vectorial capacity.

The aim of the present study was to determine the identity and diversity of blood meals taken from vertebrate hosts in wild-caught biting midges (*Culicoides* species) near livestock farms. The study includes some of the most common and most often encountered species of biting midges in Denmark; *Culicoides obsoletus* (Meigen), *Culicoides pulicaris* (L.) and *Culicoides punctatus* (Meigen), and a few of the less often encountered species in Denmark, namely, *Culicoides furcillatus* Callot, Krémer & Paradis and *Culicoides vexans* Staeger. Where other studies on this topic have collected biting midges for days or a few weeks [9,11,13], in this study a collection (20 weeks) was made that included most of the biting midge season for the same year.

## Methods

### Trapping methods

Adult *Culicoides* were captured using modified Pennsylvanian and Texas traps [17,18]. The trap consisted of a central 18 W UV fluorescent light tube mounted on top of three metal rods and fixed to a plastic funnel (25 cm in diameter). The entire unit fitted neatly into a 10 L plastic bucket. A collecting and killing jar (1 L volume) containing 70% ethanol was placed inside the bucket and below the plastic funnel. Insects attracted by the light would drop into the jar and be killed instantly. Netting (mesh size 2 mm) was used to prevent large insects from entering the trap. The light traps were set to operate from approximately 1–2 h before sunset to 1–2 h after sunrise.

### Sampling sites

All collection sites were located on the island Sealand, Denmark, which was a BTV vaccination zone in 2009. Biting midges were collected each night on weekly intervals (6–8 days) between May 8 and October 13 2009, with the exception of the last week of June (a total of 151 days), at four study sites (Table 1). Two sites were located near Knabstrup and two sites near Skellingsted approximately 13 km south-west from Knabstrup. A total of four light traps were used for the collections. One outdoor trap was located, placed on the ground, at a hobby farm with 15 cows and two horses by the village of Knabstrup. Another outdoor trap was placed 800 m away from the farm in a nearby forest on the ground close to enclosures used for raising pheasants for game. Pheasants and deer were often observed in the periods where biting midges were captured. Both trap locations were identical to those earlier described [10]. Approximately 13 km south-west in Skellingsted the other two traps were placed with a distance of approximately 500 m between them. One trap was located hanging approximately 2 m up on the wall inside a cow barn (with a high ceiling) with Angus cattle, which had access to the outdoors through several ports. The other trap was located, placed on the ground, at a hobby farm, breeding goats for slaughter, but also with other typical farm animals (free ranging hens, two dogs, two horses, and a cat) with the cattle grazing close to the property and the location of the trap.

**Table 1:** **Location of the traps in Mørkøv, Sealand, Denmark, and the number of blood-fed *Culicoides* collected**

**Village**	**Trap location (location reference)**	**GPS coordinates**	**Predominant animal species in the vicinity of the trap**	**Total number of collected *Culicoides* **	**Number of blood-fed females collected (analysed)**
Knabstrup	Pastures, on the ground (A)	55°40′25.27″ N; 11°33′14.77″ E	Cow, horse, pheasant	27,147	241 (75)
Knabstrup	Forest, on the ground (B)	55°40.50.98″ N; 11°33′04.05″ E	Pheasant, roe deer	5,554	71 (49)
Skellingsted	Pastures and farm courtyard, on the ground (C)	55°35′27.93″ N; 11°27′09.80″ E	Goat, cow, horse, poultry, dog, human	45,142	602 (124)
Skellingsted	Inside cow barn, on the wall (D)	55°35′37.83″ N; 11°27′33.10″ E	Cow	33,513	1,250 (53)

Total collection N = 111,356.

### Identification of biting midges

The entire contents of each trap were preserved in 70% ethanol and blood-fed female biting midges were sorted according to collection date and location. The head and wings were removed from the individuals and kept in individually labelled tubes with 70% ethanol for later morphological species verification. The thoraxes and abdomens (including the blood meals) of the individual females were subsequently subjected to DNA isolation in order to facilitate molecular identification of the blood meal host. Selected biting midges were furthermore subjected to molecular analysis in order to confirm morphological identifications. The characters described by [19] were applied in order to morphologically distinguish between *C. obsoletus* and *C. scoticus* Downes & Kettle.

Molecular identification of the *Culicoides* species was conducted as described by Lassen *et al.*[10]. After the wild-caught biting midges were determined morphologically to species level, the isolated DNA from selected individuals served as templates in subsequent PCR reactions. The molecular marker cytochrome oxidase I (COI barcode) was applied to identify the species of the collected biting midges [10]. Sequence alignment and phylogenetic analysis were carried out using the *Mega5* software [20]. The COI barcodes of biting midges from this study can be found as GenBank accessions JQ683259-JQ683374.

### Molecular blood meal analysis

DNA isolation from the blood-fed biting midges was carried out using the DNeasy Blood & Tissue Kit (Qiagen) [10]. The entire blood meal analyses were conducted according to the published procedure for mosquitoes [21] and the technical details described by Lassen *et al.*[10]. The samples were first screened with a species-specific primer pair for cow (*Bos taurus* L.), with the visualisation of a PCR product from any given sample in a gel electrophoresis deemed to be a positive cow result for that sample. A universal primer pair, the cytochrome b (*Cyt b)* primer pair [22], was then applied to the cow-negative samples. The purified PCR products were sequenced on a commercial basis by Eurofins-MWG|Operon (Ebersberg, Germany). The resulting FASTA files were then used for species identification according to the nucleotide–nucleotide basic alignment search tool (BLAST) in the GenBank DNA sequence database (http://www.ncbi.nlm.nih.gov/). Several samples which tested positive for cow as the blood meal host by the species-specific primer pair were further subjected to amplification with the universal primer pair, and resulting sequences were confirmed in GenBank. Samples that failed, e.g. due to sequencing difficulties or poor DNA extraction, when sequenced were analysed and sequenced again two or three times before a fail was accepted and recorded in the results.

## Results

### Identification of *Culicoides* species

Twenty-four distinct species of *Culicoides* were identified among the 111,356 biting midges collected. Besides the 20 species listed in Table 2, *C. stigma* (Meigen), *C. impunctatus* Goetghebuer, *C. grisescens* Edwards and *C. puncticollis* (Becker) were present but not found among the blood-fed specimens. The focus of the sampling was blood-fed biting midges and therefore additional non-fed specimens of other *Culicoides* species than the species listed in Table 3 could have been missed in the total collection. The species *C. pulicaris*, *C. punctatus*, *C. obsoletus* and *C. scoticus* were captured blood-fed from all four locations.

**Table 2:** **Seasonal distribution of the blood-fed biting midges ( *Culicoides* spp.) caught and analysed in the present study (n = 307)**

	**Collection week (the week number of which the traps were emptied)**																			
	**May**			**June**				**July**				**August**				**September**				
*Culicoides* species	20	21	22	23	24	25	26	28^d^	29	30	31	32	33	34	35	36^d^	37	38	39	40
*C. chiopterus*^a^		●			●									●	●		●		●	
*C. circumscriptus*^c^																	●	●	●	
*C. dewulfi*^a^		●	●			●	●				●				●			●	●	●
*C. duddingstoni*		●									●									
*C. festivipennis*^c^									●		●						●			
*C. furcillatus*											●									
*C. kibunensis*									●		●	●								
*C. lupicaris*L2		●									●									
*C. obsoletus*^a^			●		●	●	●		●		●	●	●	●	●			●	●	●
*C. obsoletus*O2			●																	
*C. pallidi-cornis*												●								
*C. pictipennis*		●	●								●									
*C. poperinghensis*		●	●	●	●															
*C. pulicaris*^b^	●		●	●		●			●		●	●		●			●	●	●	●
*C. punctatus*^b^	●		●		●	●	●		●											
*C. reconditus*			●																	
*C. riethi*																	●			
*C. salinarius*												●								
*C. scoticus*^a^		●	●		●	●	●		●		●	●	●		●		●		●	
*C. vexans*	●	●	●		●															

Collection was performed on weekly intervals (6–8 days) at four locations between May 8 th and October 6 th 2009, with the exception of the last week of June.

a) Members of the subgenus *Avaritia* were present blood-fed during the whole collection period.

b) Members of these species were present during the whole collection period, but not always blood-fed.

c) Members of these species were occasionally found during the whole collection period, but seldom blood-fed.

No blood-fed biting midges were collected in week 28, a few in week 36 but none analysed.

**Table 3:** **Mammalian blood meal host of *Culicoides* biting midges collected by UV-traps (n = 218)**

**Blood meal host/biting midge species**	** *Bos taurus* , cow**	** *Capra hircus* , goat**	** *Capreolus capreolus* , European roe deer**	** *Cervus elaphus* , red deer**	** *Equus caballus* , horse**	** *Homo sapiens* , human**	** *Mus musculus* , house mouse**	** *Ovis aries* , sheep**
*C. chiopterus*	6 ^a,c,d^	-	1^b^	-	-	-	-	-
*C. circumscriptus*	-	-	-	-	-	1 ^c^	-	-
*C. dewulfi*	9 ^c,d^	-	-	-	-	1 ^b^	-	-
*C. furcillatus*	1 ^c^	-	-	-	-	-	-	-
*C. kibunensis*	1 ^c^	-	-	-	-	-	-	-
*C. lupicaris*L2	2 ^c,d^	-	-	-	-	-	-	-
*C. obsoletus*	29 ^a,b,c,d^	5 ^c^	1 ^b^	1 ^c^	3 ^c^	3 ^b,c^	1 ^c^	1 ^a^
*C. obsoletus*O2	-	1 ^c^	-	-	-	-	-	-
*C. pallidicornis*	-	1 ^c^	-	-	-	-	-	-
*C. poperinghensis*	10 ^c,d^	-	-	-	-	-	-	-
*C. pulicaris*	42 ^a,b,c,d^	2 ^c^	-	1 ^c^	1 ^c^	-	-	-
*C. punctatus*	36 ^a,b,c,d^	1 ^c^	1 ^c^	-	-	-	-	-
*C. riethi*	1 ^d^	-	-	-	-	-	-	-
*C. scoticus*	14 ^a,b,c,d^	-	2 ^b^	-	1 ^c^	1 ^c^	-	-
*C. vexans*	35 ^a^	-	1 ^b^	-	-	1 ^b^	-	-
Total	186	10	6	2	5	7	1	1

a) *Culicoides* spp. caught by pastures at Knabstrup.

b) *Culicoides* spp. caught in a forest at Knabstrup.

c) *Culicoides* spp. caught at a hobby farm at Skellingsted.

d) *Culicoides* spp. caught inside the cow barn at Skellingsted.

Specimens of each of the identified species have been verified both morphologically and by PCR with the COI barcode sequence. Sequences for most *Culicoides* specimens in the present study have been submitted to GenBank: JQ683259-JQ683374.

Table 2 displays the seasonal distribution of the blood-fed females analysed in this study. Those blood-fed biting midges representing the most commonly captured species (*C. pulicaris*, *C. punctatus* and the four species belonging to the subgenus *Avaritia* Fox were present throughout the entire collection period, while other species exhibited a more distinct phenology; blood-fed *C. vexans* appeared in weeks 20–24 only, blood-fed *C. poperinghensis* Goetghebuer in weeks 21–24, blood-fed *C. circumscriptus* Kieffer in weeks 37–39, and blood-fed *C. kibunensis* Tokunaga in weeks 29–32 (Table 2).

Twelve *Culicoides* species were captured blood-fed from the hobby farm outside Knabstrup, and likewise twelve from the forest 800 m away (Table 3 and 4). Fifteen *Culicoides* species were captured blood-fed at the hobby farm in Skellingsted (Table 3 and 4), and ten were captured blood-fed from inside the farm in Skellingsted (Table 3).

**Table 4:** **Avian blood meal host of *Culicoides* biting midges collected by UV-traps (n = 24)**

**Blood meal host/biting midge species**	** *Acrocephalus palustris* , marsh warbler**	** *Columba palumbus* , common wood pigeon**	** *Emberiza citrinella* , yellow-hammer**	** *Garrulus glandarius* , Eurasian jay**	** *Passer montanus* , Eurasian tree sparrow**	** *Pica pica* , magpie**	** *Turdus merula* , blackbird**
*C. circumscriptus*	-	-	-	-	-	2 ^c^	1 ^c^
*C. duddingstoni*	-	-	-	1 ^b^	-	-	-
*C. festivipennis*	-	3 ^c^	-	-	-	-	-
*C. kibunensis*	1 ^c^	11 ^c^	1 ^b^	-	-	-	-
*C. pictipennis*	-	-	-	-	-	2^c^	-
*C. reconditus*	-	-	-	-	-	1 ^b^	-
*C. salinarius*	-	-	-	-	1 ^a^	-	-
Total	1	14	1	1	1	5	1

a) *Culicoides* spp. caught by pastures at Knabstrup.

b) *Culicoides* spp. caught in a forest at Knabstrup.

c) *Culicoides* spp. caught at a hobby farm at Skellingsted.

d) *Culicoides* spp. caught inside the cow barn at Skellingsted.

### Blood meals

Twenty species of *Culicoides* were represented by blood-fed females among the collected biting midges. The total number of blood-fed biting midges caught was 2,164 (Table 1), and the most common blood-fed species observed was *C. obsoletus*. Biting midges for blood meal analysis were roughly selected based on species, when present less often encountered species were preferred to those present in high numbers throughout the collection.

Blood meal sources were successfully identified by DNA sequencing in 242 (76%) out of 320 *Culicoides* specimens (Table 3 and 4). No mixed blood meals were observed. Fifteen vertebrate species, including both mammals and birds, were identified as hosts, as listed in Table 3 and 4. The most common host species was cow (*B. taurus*), which constituted 77% of the identified blood meals (Table 3). The second most numerous host species was common wood pigeon (*Columba palumbus* L.), which constituted 6% of the identified blood meals (Table 4). All other blood meal hosts listed in Table 3 and 4 were present at a rate of ≤2% of the identified blood meals (Table 3 and 4).

Of the blood-fed biting midges analysed by PCR, 49 had been caught in the forest (Table 1), and identification of the blood meal host failed to give a matching sequence in 29 cases (59%). Of the 20 blood meals that were identifiable 11 had fed on cow, three on roe deer *(Capreolus capreolus* L.), three on human (*Homo sapiens* L.) (Table 3), and one on magpie (*Pica pica* L.), yellowhammer (*Emberiza citrinella* L.), and Eurasian jay (*Garrulus glandarius* L.), respectively (Table 4). In particular, blood meals from *C. punctatus* and *C. pictipennis* Staeger exhibited a tendency to fail in molecular blood meal identification. Furthermore, there were difficulties with identifying the blood meal host of *C. punctatus* captured at the hobby farm in Skellingsted as 39% of the 33 blood-fed *C. punctatus* attempted failed consistently despite a number of attempts. In contrast, all 53 blood meals from inside the cow barn, but one, were identified, and all were cow.

Seven different birds were found as blood meal hosts, with the most prevalent being common wood pigeon (Table 3). In total, 5.8% of the blood meals originated from birds (n = 18). Fourteen of these were caught at the hobby farm near Skellingsted. Nine ornithophilic *Culicoides* species were represented in this study with blood meals from birds only (Table 4). The only two biting midge species to have fed from both birds and mammals were *C. kibunensis* and *C. circumscriptus* (Table 3 and 4).

The majority of *C. pulicaris* specimens had fed on cow (n = 42), but goat (*Capra aegagrus hircus* L.) (n = 2), horse (*Equus caballus* L.) (n = 1) and red deer (*Cervus elaphus* L.) (n = 1) were also identified as blood meal hosts (Table 3). *C*. *obsoletus*, *C. scoticus, C. chiopterus*, *C. dewulfi* Goetghebuer, *C. punctatus*, *C. vexans* and *C. poperinghensis* had likewise fed only on mammals and primarily on cow (Table 3). *C. obsoletus* was the only species to have fed, at least once, on all the identified mammalian blood meal hosts, including the only identified blood meals from sheep (*Ovis aries* L.) and mouse (*Mus musculus* L.), (Table 3).

## Discussion

The most numerous *Culicoides* species collected in this study belonged to either of the subgenera *Avaritia* (*C. obsoletus* group) or *Culicoides* (*C. pulicaris* group), which is in agreement with other collections in Europe made with UV-light traps [9,11,23-25]. Correct identification of field-collected specimens to species level, rather than just complex level, is essential in order to understand the role of *Culicoides* in the transmission cycle of pathogens. This is reinforced by the differences in blood meal host choice of *C. obsoletus* and the other members of subgenus *Avaritia* (*C. chiopterusC. dewulfi and C. scoticus*) (Table 3). *C. obsoletus* is evidently less restricted in its blood meal choice than the rest.

The blood-fed biting midges belonging to the most commonly captured species were chosen for molecular analysis in a random manner, as their large number made it impossible to analysis them in full in a reasonable time. The blood-fed biting midges belonging to the less commonly encountered species e.g. *C. furcillatus*, *C. riethi* Kieffer and *C. poperinghensis* were all subjected to sequencing. Eleven *Culicoides* species are represented in the study by three or less specimens (Table 3 and 4). Due to the large number of *Culicoides* biting midges collected during this study (n = 111,356), our particular focus was restricted to blood-fed females only. Thus, Table 1 gives a somewhat biased representation of the seasonal distribution of Danish biting midges as the less common species may be present in more weeks than the one or two weeks a blood-fed specimen was caught and recorded.

*C. vexans* is apparently appearing in late spring only. At the hobby farm near Knabstrup we collected a surprising large number of *C. vexans*. The species is seldom encountered in Denmark in large numbers. Of the 30 engorged *C. vexans* females analysed 29 had fed on cows and 1 had fed on a human (Table 3). Jobling [26] has reported *C. vexans* as being “a fierce man-eater”, but when human hosts are scarce *C. vexans* do not have difficulties in feeding from another host. Our collection indicates that *C. vexans* is mostly prevalent in mid-May and has only one population peak (Table 1). *C. vexans* has been recorded to have a single population peak per year around London, though at an earlier time in the year than we report here [26]. It is therefore likely that *C. vexans* has one egg-laying cycle, and consequently takes one blood meal only, per generation. Until now, *C. vexans* has not been included in studies regarding transmission risk of BTV and in agreement with this the single peak we find here suggests that the risk of *C. vexans* transmitting BTV, or any other virus, may be low.

Repeated findings in the present and a previous study [10] with some overlap in methods and location add significance to the observations, e.g. biting midges with cow blood in the forest trap. At the same trap location in the forest near Knabstrup as the previous year [10] we collected biting midges that had fed on cows 800 m away from the nearest cow. Blood meals from eight females belonging to five *Culicoides* species were identified as being of cow origin. This strongly suggests that they have somehow dispersed into the forest after blood-feeding, presumably at the nearest hobby farm. Five of these engorged females were caught in late September, which is the same time of year that the cow blood meal from the preceding year was caught.

Biting midge flight is limited but they may travel long distances with the prevailing wind [27,28]. The biting midges may have been carried away by wind from the immediate area after feeding, while leaving the blood meal host in order to find somewhere to digest. Biting midges spend up to 90% of their time digesting blood meals and developing eggs [29]. Furthermore, it cannot be excluded that they travelled a much longer distance than first supposed, as it has been indicated that biting midges can be dispersed over large distances by atmospheric transportation e.g. from Germany to the south of Sweden [30]. The one blood meal originating from a sheep (Table 3) is another example of blood-fed biting midges travelling long distances after their blood meal. No sheep was closer than approximately 1.5 km from where the blood meal was collected (trap location A, Table 1).

This extended collection period as compared to 2008 made a difference between the number of *Culicoides* species identified in 2009 (n = 24) compared to 2008 (n = 9). The results listed on Table 3 and 4 are valuable for assessing the extent of contact between *Culicoides* biting midges and different hosts. They do no not, however, say anything of the dynamic feeding behaviour of biting midges in response to host availability [31]. In the present study, the mammalian species which was most frequently identified as a host of biting midges was the cow (Table 3). This observation is expected given that cow was the most common host available in the vicinity of three out of the four trap locations. In addition, a preliminary study has already suggested cow as the most common blood meal host [10].

In contrast to the preceding year, horse was not found to be the blood meal host of engorged biting midges caught at the hobby farm in Knabstrup [10]. The availability of horses in the vicinity of the trap was the same for both years. In fact, every location had cow as the most numerous blood meal host, and even the trap in the forest caught a higher number of specimens with blood meals originating from cow than of any other potential hosts, and yet no cow was nearer than 800 m.

Four species (*C. obsoletusC. scoticusC. pulicaris* and *C. poperinghensis*) caught in the forest were found to have fed on both cow and deer. Of these four species *C. obsoletus* and *C. scoticus* have been proven to be able to be infected with BTV [32,33]**.** This overlap in blood meal hosts makes it possible to introduce BTV to the wild deer population in the area, should there be BTV positive cows at the hobby farm or vice versa. This also applies for the hobby farm near Skellingsted where *C. obsoletus* and *C. pulicaris* were found to have fed from mostly cow but in one instance each on a red deer. In a hypothetical worst case scenario this could lead to the establishment of a BTV reservoir outside the range of possible vaccination programs. Although no conclusion can be made about the dynamic feeding behaviour of biting midges in response to host availability, it is apparent that cow is chosen as the primary blood meal host. It is not possible at this time to speculate about whether cow would still have been preferred if any other vertebrates had been available, but as cattle is the most numerous host on some farms in Denmark the potential host options for blood feeding on other species is limited.

The traps were placed on the ground as biting midges feeding from mammals were the main target in this study. However, some biting midges were found to have fed exclusively on birds (Table 4). Birds as blood meal hosts in the context of BTV transmission is a dead end. The low number of blood meals originating from birds in this study may be explained by the placement of the traps on or near the ground. Cerný *et al.*[13] had success with capturing *Culicoides* by using special nest boxes occupied by live wild birds (bird-baited traps about 3 m from the ground at canopy level. According to Cerný *et al.*[13]*C. kibunensisC. festivipennis* Kieffer and *C. circumscriptus* preferred the canopy level, and thus suggested these species to be ornithophilic. *C. pictipennis* was also suggested to prefer the canopy level but this remains to be proven [13]. Our results are largely in agreement with these published findings. Our traps at ground level collected relatively few, compared with e.g. *C. pulicaris* or *C. obsoletus*, of those biting midge species. The *C. kibunensisC. festivipennis* and *C. circumscriptus* that were captured had fed on birds, with the exception of single *C. circumscriptus* and *C. kibunensis* (Table 3 and 4), which had fed on human and cow, respectively.

*Culicoides* feeding on a hen (*Gallus gallus domesticus* Brisson) or pheasant (*Phasianus colchicus* L.) were not observed in the present study, despite the fact that pheasants were observed at trap locations A and B, and hens were running free at trap location C. This agrees with the earlier published findings from Lassen *et al.*[10]. This may be due in part to the behaviour of hens and pheasant, as they rest in trees in the active hours of the biting midges, in combination with our traps being placed on the ground.

Ten *Culicoides* species are represented in the study by five or less blood-fed females with identified blood meal hosts (Table 3 and 4). Thus, it is difficult to say what their host preference might be. The low catches may be due to placement of the traps on the ground. Blood-sucking flies may choose a particular level above ground and feed on a range of hosts encountered at that level [34,35]. Swanson *et al.*[36] observed that the largest catch of *C. pictipennis* was at 10 m above the ground in a spruce forest and was thus associated with avian hosts. The low catch of blood-fed *C. pictipennis* (n = 9) in this present study by traps at ground level and the identified blood meals from magpie (n = 2) suggests a similar association. Swanson *et al.*[36] also observed a relatively high number of *C. salinarius* Kieffer specimens (n = 15) near ground level in a pine forest, and therefore associated this species with mammal hosts. We caught a single blood-fed *C. salinarius* near the ground at the hobby farm near Knabstrup that had fed from a tree sparrow (*Passer montanus* L.).

Contrary to what might have been expected based on earlier findings [10], less *Culicoides* species were found feeding from both birds and mammals. The only two species to feed from both birds and mammals were *C. kibunensis* and *C. circumscriptus* (Table 3 and 4). In the preceding year *C. obsoletus, C. scoticusC. chiopterus* and *C. punctatus* were found to have fed several times from common wood pigeons, and a few times from mallards (*Anas platyrhynchos* L.) [10]. We would have expected these four species to feed at least a few times on common wood pigeons as in the autumn of the previous year at the same locations, where many birds of different species were observed [10]. It is tempting to assign a *Culicoides* species to be either ornithophilic or mammalophilic. However, in collections for two consecutive years six out of 20 species had fed on both birds and mammals, though in all cases there seems to be a preference for either mammals or birds, e.g. *C. punctatus* which had fed from a bird, rather than a mammal, in eight out of 59 blood meals [10]. Feeding from more than one type of host facilitates transmission of virus to incidental hosts, as in the case of West Nile Virus and the vector *Culex quinquefasciatus* Say [37], which makes *Culicoides* biting midges potential bridge vectors.

Although *C. obsoletus* are the most varied in their blood meal host choice, they appear in this study to be strictly mammalophilic, in contrast with the findings from the subsequent year [10], where *C. obsoletus* had fed from a bird in seven cases (n = 7). In fact, none of the members of subgenus *Avaritia* present in this study were found to have fed on a bird, while five to seven specimens from each species had done so the preceding year [10]. This is surprising when one considers that the collection period the preceding year was 5 weeks during the autumn [10], and this study consists of catches from May to October. Host availability plays an important role in feeding behaviour of biting midges in general; however, further studies are needed to determine the exact role of all contributing factors to the variation in host feeding patterns among the different *Culicoides* species.

The rate of successful identification of blood meals in this study was comparatively low (76%, n = 307). In a preceding study it was 90% (n = 128) [10]; Ninio *et al.*[9] had one of 91% (n = 157); Garros *et al.*[12] had one of 92% (n = 141); and Molaei and Andreadis [21] had one of ≈ 100%. About 24% of the blood meal analyses in this study failed to result in a vertebrate blood meal host. Only few samples failed completely, which could be explained by the possibility that the blood was too degraded to yield a vertebrate PCR amplification product. Most of the failed samples yielded ample amounts of COI PCR product, but did not match any vertebrate in the GenBank database. Thus, the blood meal might not originate from a vertebrate. Some studies [38-41] have proven that mosquitoes (*Aedes* sp.) in captivity are attracted to and feed on insect larvae, and live to produce viable eggs [39]. Moreover, other genera belonging to Ceratopogonidae are known to feed on the haemolymph from insects [42]. Thus, the hypothesis that *Culicoides* do the same when in need of protein may not be improbable. This is further supported by the apparent pattern of locations from where the blood meals were collected. No failed blood meals came from the cow barn near Skellingsted, while the three outdoor locations resulted in 19 to 30 failed blood meals each. This may be due to several factors, with one likely to be the fluctuating availability of vertebrate host in the vicinity of the trap. In contrast, there was always a cow close to the indoor trap at the cow barn. The possibility of invertebrate meals should be considered and investigated further.

## Conclusions

Our results suggest that some *Culicoides* species are opportunistic and readily feed on a variety of mammals and birds, while the rest seems to be strictly mammalophilic or ornithophilic. Based on their number, dispersal potential and blood feeding behaviour, we conclude that *Culicoides* biting midges are potential vectors for many pathogens not yet introduced to Denmark.

Further research is needed to determine the ability of *Culicoides* to transmit various pathogens now present in subtropic and tropic regions in a temperate climate e.g. as was the case with BTV and Schmallenberg virus.

The development of a method combining barcoding and e.g. pyrosequencing will make it possible to analyse up to several thousand *Culicoides* specimens including blood meals in a single analytic step and would give a new basis for assessing vector-host interactions and predict vector potential.

## Competing interests

The authors declare that they have no competing interests.

## Author’s contributions

This is part of a PhD project at the Aarhus University Graduate School of Science and Technology. SBL and MK conceived the study. SBL carried out the field work and designed and performed the lab experiments supervised by MK. SBL and SAN identified *Culicoides* specimens. SBL analysed the data, interpreted the results and wrote the first draft of the paper. All authors read and approved the final manuscript.
